# A Rare and Intriguing Case Report of Metaplastic Breast Carcinoma

**DOI:** 10.7759/cureus.56619

**Published:** 2024-03-21

**Authors:** Vallal Kani, Vimal Chander, Sulochana Sonti, Sridevi Manian, Sudha Vasudevan, Muthuvel Esakki, Sarah Grace Priyadarshini, Karthika Rajendran

**Affiliations:** 1 Department of Pathology, Saveetha Medical College and Hospital, Saveetha Institute of Medical and Technical Sciences, Saveetha University, Chennai, IND

**Keywords:** metaplastic breast carcinoma, chemotherapy, hormone receptor, triple-negative, mesenchymal

## Abstract

Metaplastic breast carcinoma (MBC) is a rare and aggressive subtype of breast cancer characterized by the presence of both epithelial and mesenchymal components within the tumor. Its clinical and radiological appearance is comparable to other types of breast cancer, but it grows rapidly. The diagnosis of metaplastic carcinoma is largely based on the epithelial origin of the cells confirmed by immunohistochemistry (IHC). Compared to invasive ductal carcinoma, metaplastic carcinoma has a worse overall survival rate. Any patient with a rapidly growing breast mass should be assessed with suspicion of sarcomatoid or metaplastic malignant neoplasm. We report this case due to its rarity and the complex nature of the disease.

## Introduction

Metaplastic breast carcinoma (MBC) stands as a rare and distinct aggressive subtype of breast cancer, characterized by its unique histological features and challenging clinical management. Its incidence is 0.2-5% of all cancers of the breast and has the worst prognosis when compared to other forms of the disease, leading to the major cause of mortality due to breast carcinoma globally [1]. In 1973, Huvos and associates published the first publication introducing the term metaplastic carcinoma [1,2]. In this article, we report a case of metaplastic breast carcinoma in a 44-year-old female.

## Case presentation

**Figure 1:**
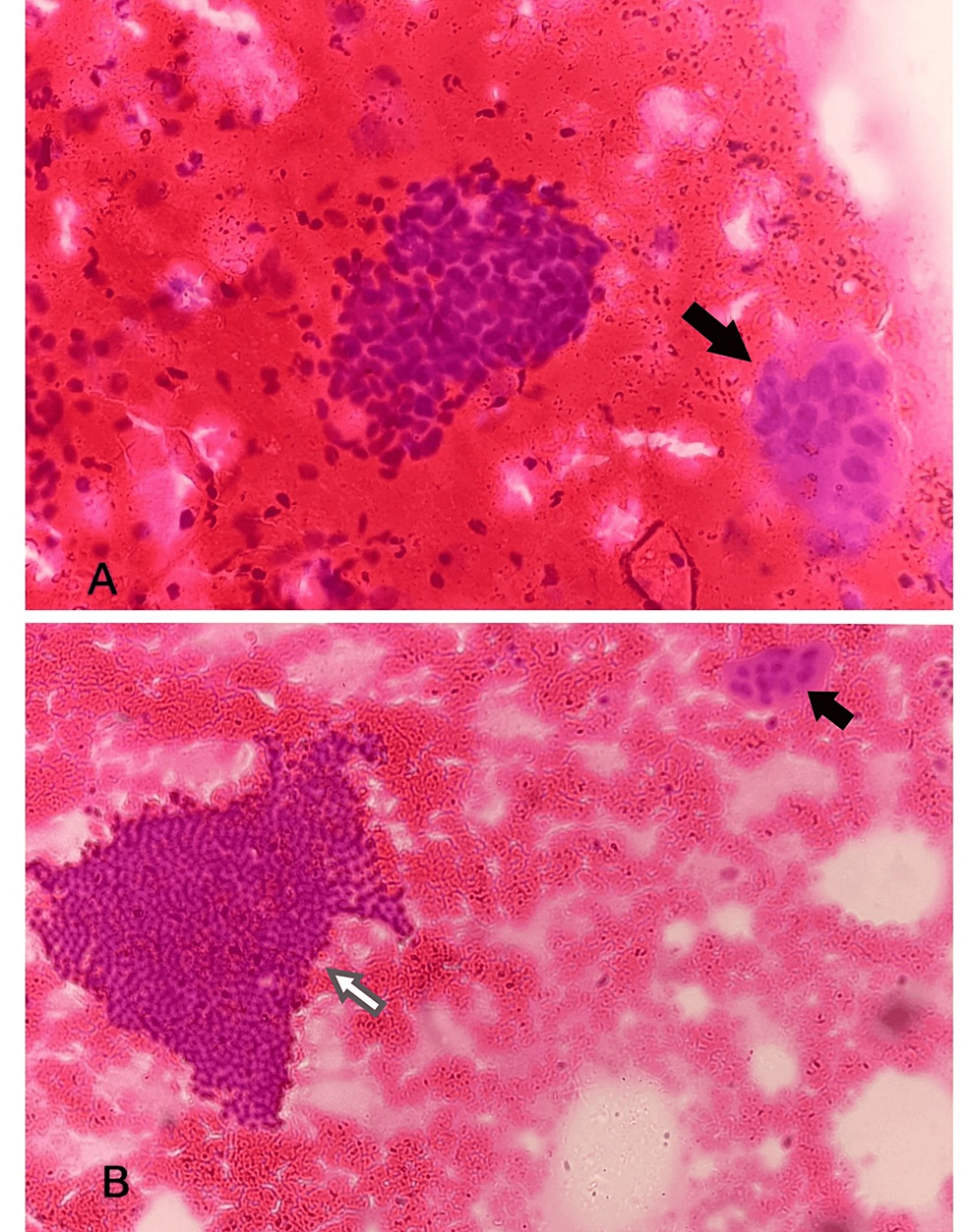
Fine needle aspiration cytology A: Smear showing ductal epithelial cells and multinucleated giant cells (arrow); B: Monolayered sheets of ductal epithelial cells (white arrow) with multinucleated giant cells (black arrow)

A 44-year-old female has been complaining of a lump in the right breast for the past four months, associated with pain. On examination, a 7x6 cm swelling was noted in the right breast, and with a clinical diagnosis of fibroadenoma, fine needle aspiration and trucut biopsy followed by an excision biopsy were performed. In fine needle aspiration cytology (FNAC), the smears were moderately cellular and showed branching papillary fronds, monolayered sheets, clusters of ductal epithelial cells with interspersed myoepithelial cells, a few bare nuclei, occasional clusters of apocrine cells, multinucleated giant cells, and a few anucleate squames in a background of hemorrhage. The smears studied showed no evidence of malignancy (Figures 1A, 1B). Hence, a diagnosis suggestive of fibroadenoma with focal foreign body giant cell reaction was given.

**Figure 2:**
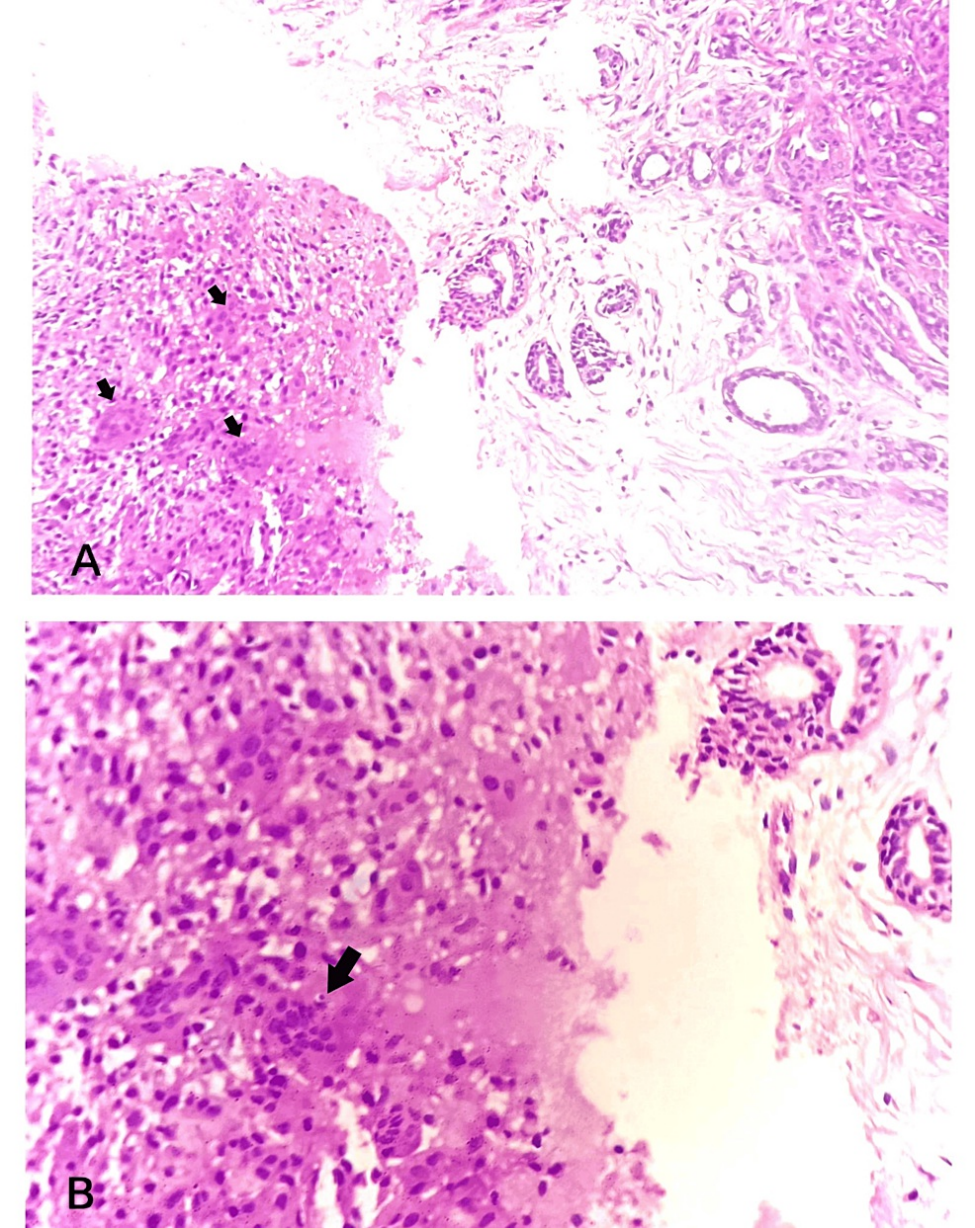
Trucut biopsy A: Sections from trucut biopsy showing glands with multinucleated giant cells (arrows); B: Sections from trucut biopsy showing multinucleated giant cells (arrow)

In a subsequent trucut biopsy, we received multiple grey-white soft tissue fragments on aggregate measuring 0.8x0.5x0.2 cm, which microscopically showed breast parenchyma with glands lined by bilayered epithelium with focal areas of foreign body giant cell reaction. A few ducts showed epithelial hyperplasia without atypia. The sections studied showed no atypical cells or granulomas (Figures 2A, 2B). The report was given as features suggestive of benign breast disease with foreign body giant cell reactions.

**Figure 3:**
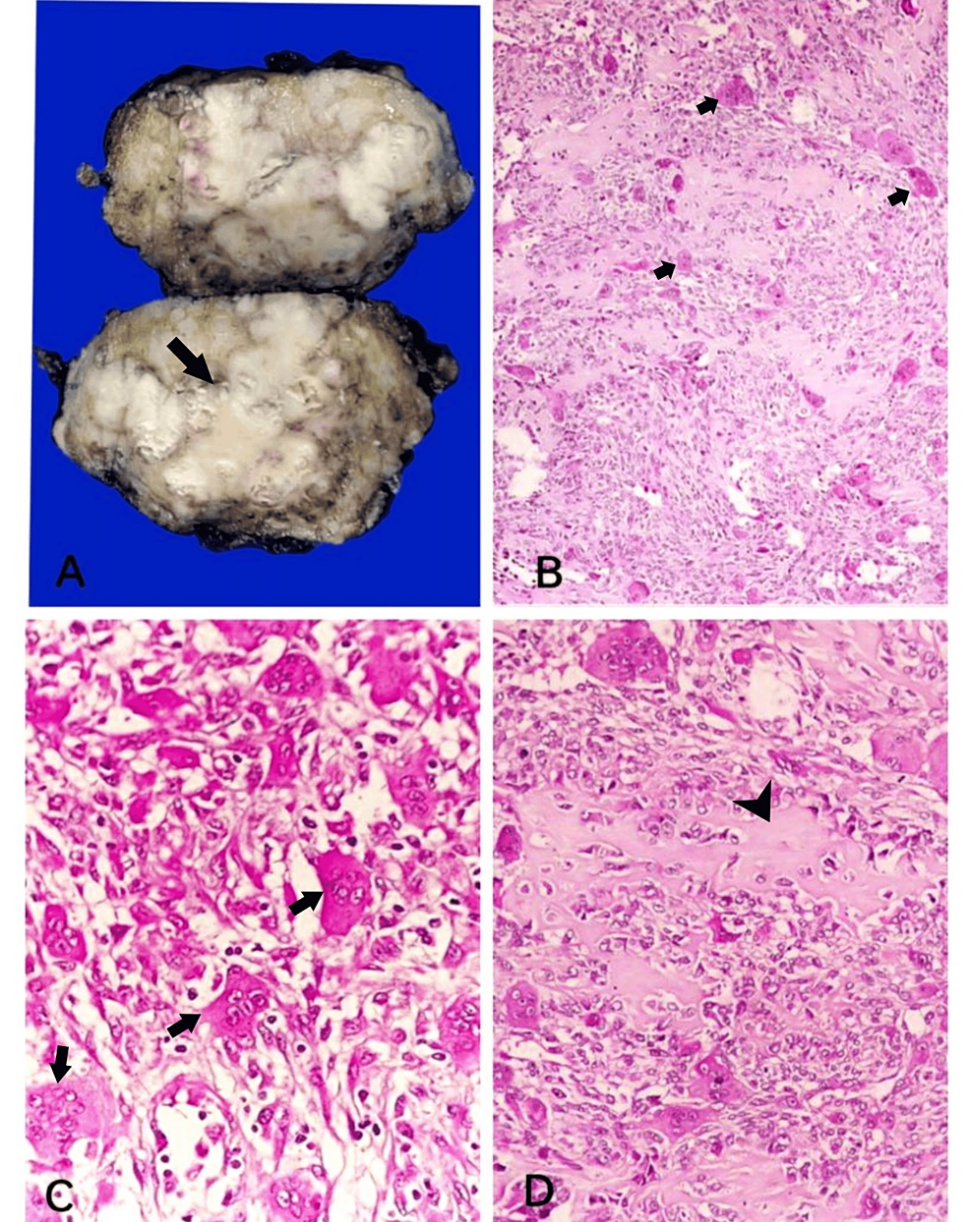
Gross and microscopy of excision biopsy A: Gross specimen showing a globular mass with a grey-white cut surface (arrow); B: Breast parenchyma with an infiltrating malignant neoplasm and numerous multinucleated giant cells (arrows); C: Elongated to spindle-shaped tumor cells with numerous scattered osteoclastic types of multinucleated giant cells (arrows); D: Areas of eosinophilic osteoid (arrowhead) separating the tumor cells

**Figure 4:**
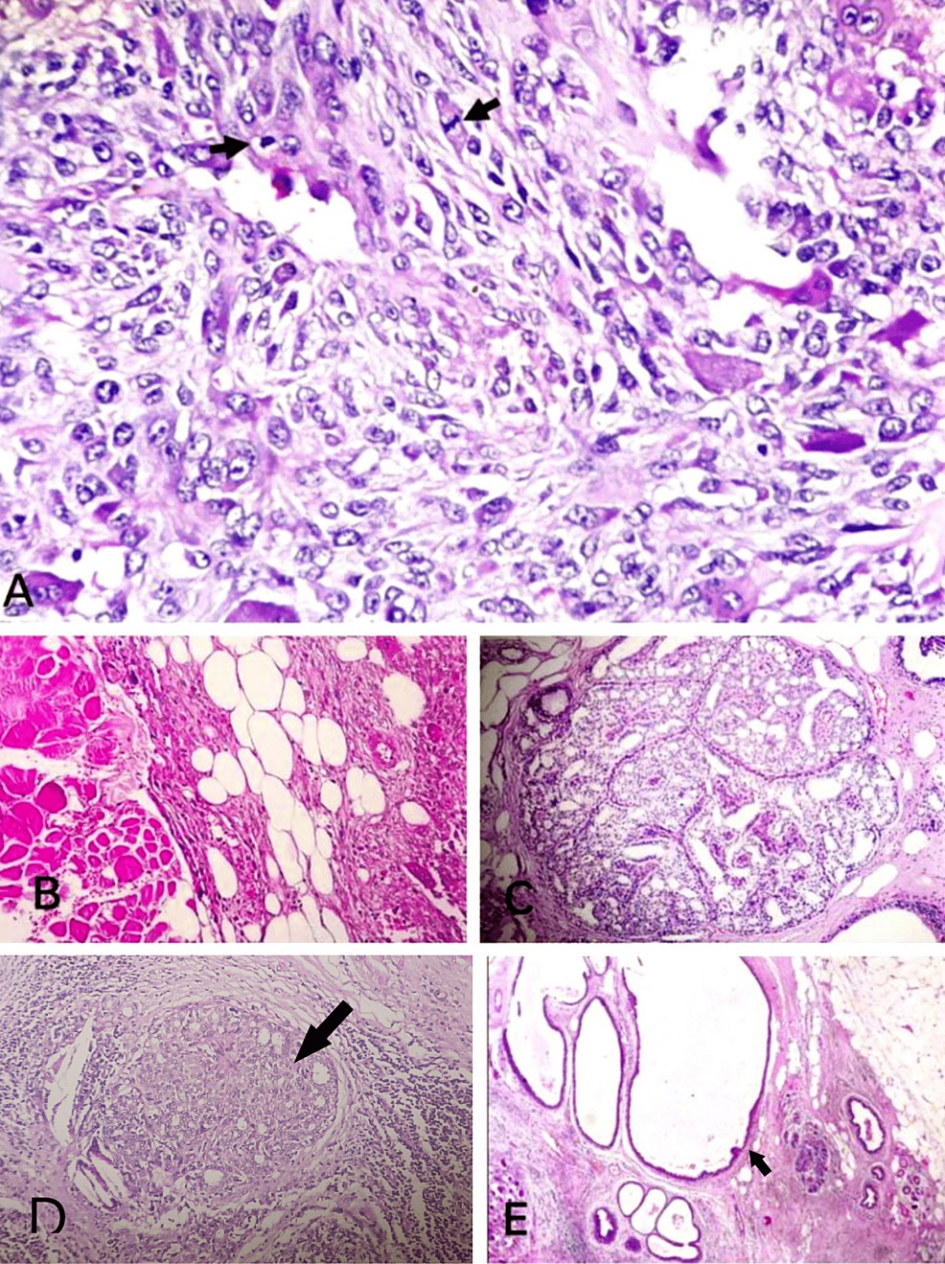
Microscopy A: Mitotic figures (arrows); B: Tumor cells invading the adipose tissue and adjacent breast parenchyma; C: Areas of usual ductal hyperplasia; D: Ductal carcinoma in situ component (arrow); E: Areas of fibrocystic change (arrow)

**Figure 5:**
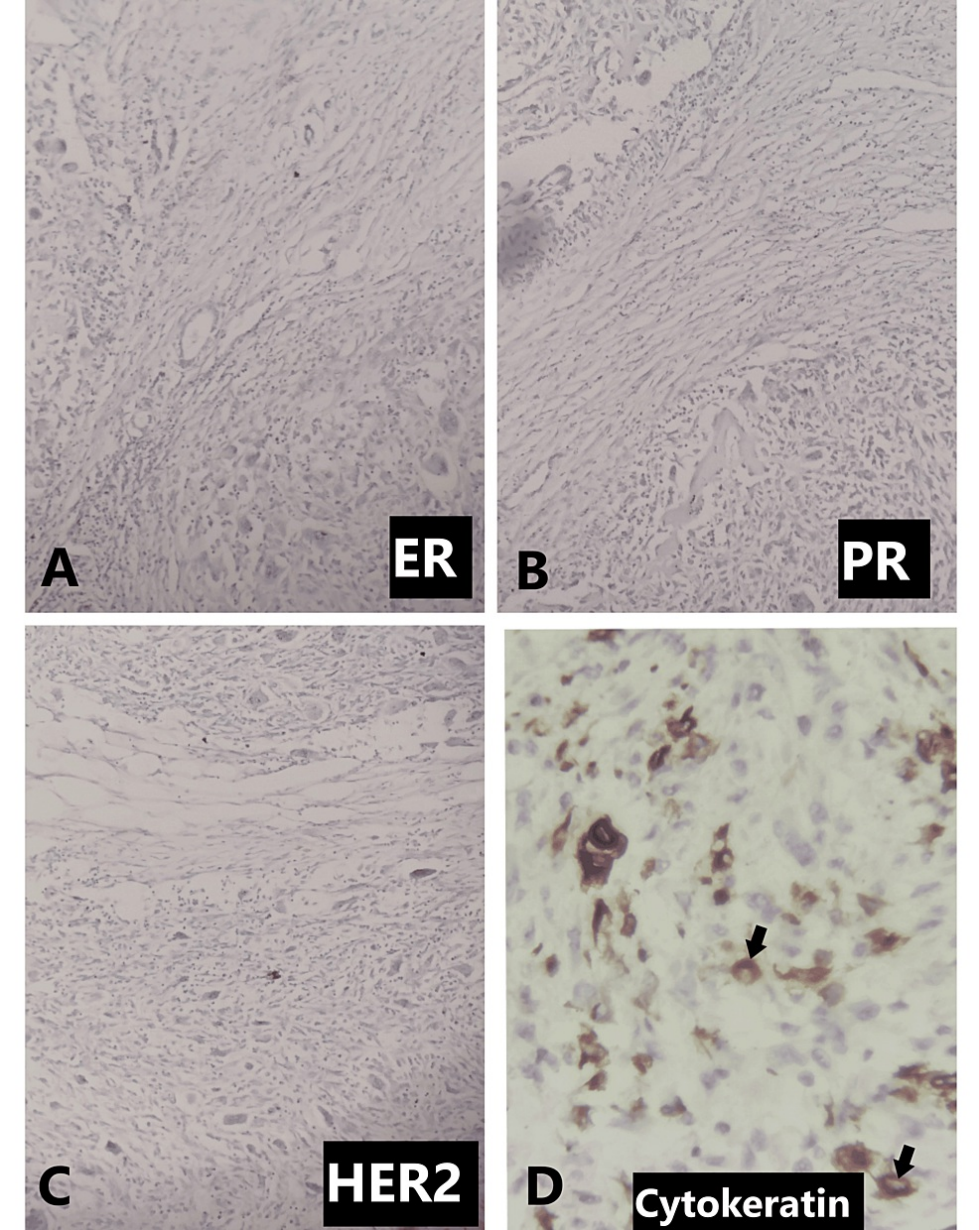
Immunohistochemistry A: Immunohistochemistry showing ER negativity; B: Immunohistochemistry showing PR negativity; C: Immunohistochemistry showing HER2 negativity; D: Immunohistochemistry showing 40% cytokeratin positivity (arrows)

Then an excision biopsy was done, and macroscopically, we received a cut-open single globular mass measuring 8x5x4 cm, and the cut surface showed grey-white to grey-brown areas (Figure 3A). On microscopy, multiple sections studied showed breast parenchyma with an infiltrating malignant neoplasm composed of sheets of cells with scant to moderate eosinophilic cytoplasm and elongated to spindle-shaped hyperchromatic, pleomorphic nuclei with brisk mitosis, along with numerous scattered osteoclastic types of multinucleated giant cells (Figures 3B, 3C). Focal areas showed eosinophilic osteoid separating the tumor cells along with necrosis and chondroid areas (Figure 3D). Numerous mitotic figures were noted (Figure 4A). The tumor cells were seen to invade the adipose tissue and adjacent breast parenchyma and skeletal muscle bundles in one focus (Figure 4B). Adjacent parenchyma showed dense hyalinization and fibrocystic change with focal usual ductal hyperplasia and low-grade ductal carcinoma in situ (Figures 4C-4E). Immunohistochemistry showed estrogen receptor (ER), progesterone receptor (PR), and human epidermal growth factor 2 (HER2) negativity with 40% positivity of cytokeratin (Figures 5A-5D). Based on the above findings, a final diagnosis of metaplastic carcinoma (with predominant mesenchymal/osteosarcomatous patterns) was given. No lymph nodes were submitted.

Then, an oncology opinion was obtained, which advised chemotherapy for eight cycles and planned revision surgery with a modified radical mastectomy. The patient was followed up, and she has completed two cycles of chemotherapy with Adriamycin and Cyclophosphamide and improved symptomatically.

## Discussion

Metaplastic carcinomas are defined as including one or more populations of cells that have undergone metaplastic differentiation, or the transformation of glandular morphology into non-glandular morphological forms [3]. The metaplastic alterations consist of either carcinomatous (squamous) or sarcomatous (spindle, chondroid, and osseous) components [1]. Metaplastic breast carcinoma is classified as low-grade tumors, which include low-grade adenosquamous carcinoma and fibromatosis-like spindle cell carcinoma, followed by high-grade tumors, which include high-grade adenosquamous carcinoma and aggressive high-grade spindle cell carcinoma. Rarely intermediate-grade tumors, which include spindle cell metaplastic carcinoma, are also seen, as they behave with intermediate risk between low-grade and high-grade tumors. Two primary types of metaplastic breast carcinoma are observed in practice: tumors having both metaplastic and adenocarcinomatous elements that can overlap morphologically and tumors having specific metaplastic components (such as squamous, spindle cell, or matrix-producing tumors) [4]. The major reasons for false negative results in fine needle aspiration cytology and trucut biopsy are inappropriate sampling procedures, the incorrect site of the tumor, or the existence of a well-defined neoplasm with low atypia [5]. Most metaplastic breast carcinomas do not express the human epidermal growth factor 2 receptor (HER2), progesterone receptor (PR), or estrogen receptor (ER). Hence, they fall under triple-negative breast cancer (TNBC). Regretfully, the prognosis for metaplastic breast carcinoma is poorer than that of non-metaplastic triple-negative breast cancer; it has a twofold chance of recurrence and a lower overall and disease-free life [1]. The epithelial origin of metaplastic carcinoma is mostly dependent on demonstrating a positive expression of cytokeratin, at least focally on immunohistochemistry. It also stains for vimentin and smooth muscle actin [6,7]. Because metaplastic carcinoma is uncommon and heterogeneous, the best course of treatment has not been determined. Usually, treatment for it is comparable to that for a standard case of breast adenocarcinoma. In most studies, treatment consists of modified radical mastectomy and dissection of axillary nodes, together with chemotherapy and radiotherapy. The role of targeted therapy and conventional chemotherapy is limited by the lack of hormone receptor expression and the human epidermal growth factor 2 receptor (HER2). While post-mastectomy radiotherapy was linked to advantages in overall survival in the advanced stage but not in the early stage, post-lumpectomy radiotherapy was associated with greater overall survival in both the early and advanced stages of the carcinoma [6,8]. The significant predictors of survival include the duration of symptoms, stage of the tumor, size of the tumor, and status of axillary nodes [6].

## Conclusions

This rare case report of metaplastic breast carcinoma underscores the importance of comprehensive diagnostic evaluation and individualized treatment approaches in managing uncommon breast malignancies. Despite its rarity, clinicians must maintain a high index of suspicion for metaplastic breast carcinoma when encountering atypical, rapidly growing breast lesions, especially in older patients with unique clinical presentations. Additionally, this case highlights the significance of multidisciplinary collaboration involving oncologists, pathologists, radiologists, and surgeons to ensure accurate diagnosis, optimal treatment planning, and favorable patient outcomes. Continued research efforts are warranted to further elucidate the pathogenesis, prognostic factors, and therapeutic strategies for this challenging subtype of breast cancer. Through diligent clinical observation, meticulous pathological assessment, and tailored therapeutic interventions, we can strive to improve our understanding and management of metaplastic breast carcinoma, ultimately enhancing the quality of care and outcomes for affected individuals.
